# Small Trafficking Inhibitor Retro-2 Disrupts the Microtubule-Dependent Trafficking of Autophagic Vacuoles

**DOI:** 10.3389/fcell.2020.00464

**Published:** 2020-06-18

**Authors:** Valérie Nicolas, Vanessa Lievin-Le Moal

**Affiliations:** ^1^Université Paris-Saclay, Institut Paris-Saclay d’Innovation Thérapeutique (IPSIT), Microscope Facility (MIPSIT), UMS–US31–UMS3679, Châtenay-Malabry, France; ^2^University Paris-Saclay, Inserm, UMR-S 996 Inflammation, Microbiome and Immunosurveillance, Clamart, France

**Keywords:** autophagy, nutrient starvation, microtubules, vacuolar trafficking, autophagosome, autolysosome, trafficking inhibitor Retro-2

## Abstract

Autophagy is a catabolic recycling process by which a cell degrades its own constituents to contribute to cell homeostasis or survival. We report that the small trafficking inhibitor Retro-2 impairs microtubule-dependent vacuolar trafficking in autophagy. Retro-2 induced autophagy and promoted the dramatic cytoplasmic accumulation of large autophagosomes. Moreover, Retro-2 decreased the spreading of autophagosomes within the cytoplasm of nutrient-starved cells. In addition, Retro-2 abolished autolysosomes formation. We show that these effects arise from hitherto unsuspected disassembly activity of the small molecule on the cellular microtubule network, which is known to act as a key regulator of vacuolar trafficking of the autophagy pathway.

## Introduction

Our understanding of how the autophagy pathway (Takeshige et al., 1992) is structurally and functionally regulated has evolved considerably over the last 20 years (Ohsumi, 2014). Macroautophagy (referred to as “autophagy” hereafter) is a complex and highly regulated intracellular degradation process pivotal to maintaining cellular homeostasis, as it mediates the bulk clearance of long-lived and aberrant proteins and defective organelles (Klionsky and Codogno, 2013). Moreover, autophagy has multiple effects on immunity and is involved in various human diseases including neurodegenerative diseases, infectious diseases, and cancer (Jiang and Mizushima, 2014). Autophagy starts by the formation of a membrane cisterna, called the phagophore or isolation membrane. After closure of the phagophore membrane, the formed cargo-containing autophagosome travels for controlled vacuolar fusion events, including the fusion of autophagosomes with late endosomes (LEs) and acidic lysosomes to form late degradative autolysosomes, in which autophagosome-containing cargo is enzymatically degraded to produce products that are exported back into the cell cytoplasm for reuse (Kaur and Debnath, 2015).

Retro-2 a small-molecule inhibitor identified by a cell-based high-throughput screen (Stechmann et al., 2010; Barbier et al., 2012), exerts a broad-spectrum activity against the cellular trafficking of toxins, intracellular bacterial pathogens and a large variety of viruses (Gupta et al., 2014). In this study, we aimed to decipher the impact of Retro-2 on autophagic vacuolar trafficking.

## Results

### Retro-2 Promotes Autophagy

**FIGURE 1:**
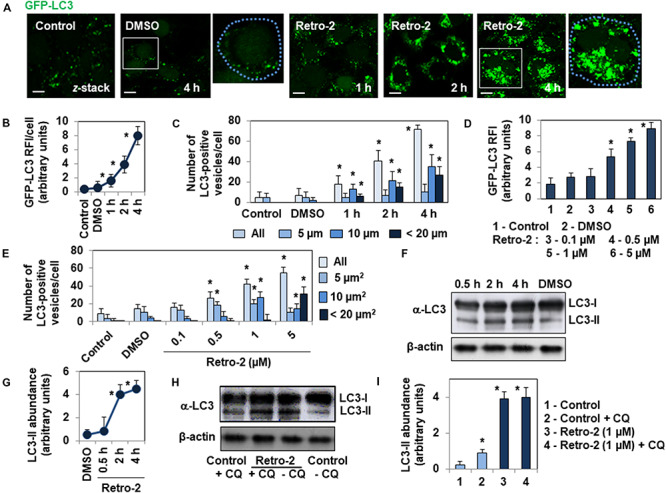
Retro-2 induces autophagy in GFP-LC3-expressing HeLa cells. **(A)** Representative CLSM micrographs showing the rare presence of GFP-LC3-positive vesicles in control and DMSO-treated cells, and the increase of GFP-LC3-positive vesicles in the cytoplasm of cells treated with Retro-2 (1 μM) during a time-course. **(B)** Graph showing the increase of the Relative Immunofluorescence Intensity (RFI) of GFP-LC3 per cell in cells treated with Retro-2 (1 μM) during a time-course. **(C)** Bar graph showing the increase of total, small (5 μm^2^), large (10 μm^2^), and very large (<20 μm^2^) GFP-LC3-positive vesicles in cells treated with Retro-2 (1 μM) during a time-course. **(D)** Bar graph showing the concentration-dependent increase of the GFP-LC3 RFI/cell in cells treated with increasing concentrations of Retro-2 for 4 h. **(E)** Quantification of all, small (5 μm^2^), large (10 μm^2^), and very large (<20 μm^2^) GFP-LC3-positive vesicles, showing their concentration-dependent increase in cells treated with increasing concentrations of Retro-2 for 4 h. **(F)** A representative Western blot showing LC3 processing in control cells and cells treated with Retro-2 (1 μM) during a time-course. β-actin was monitored to ensure equal loading of the lanes. **(G)** Graph showing the quantification of abundance of LC3-II protein determined by Western-blot analysis, showing the time-dependent increase of the autophagy marker in Retro-2 (1 μM)-treated cells during a time-course. **(H)** A representative Western blot showing the abundance of LC3-II protein in control cells and cells treated with Retro-2 in the presence, or not, of chloroquine (CQ). **(I)** Graph showing the quantification of abundance of LC3-II protein determined by Western-blot analysis, showing the equal abundance of LC3-II protein in cells treated with Retro-2 in the presence, or not, of CQ. CLSM micrographs are representative of three separate experiments in duplicate. Scale bar, 10 μm. White boxed regions delineate the areas shown in adjacent high-magnification images. Dotted blue line delineated the cell surface. The Western blots are representative of two or three separate experiments in duplicate. Western blot quantification was performed using ImageJ software. Quantification of confocal images was performed by examining at least 30 cells for each timepoint and condition using ImageJ software. Values represent the average of three separate experiments in duplicate (±SEM). Data were analyzed using the unpaired Student *t*-test. *P < 0.01 compared to Control **(B–E,G,I)**.

We evaluated the impact of Retro-2 on autophagy using HeLa cells stably expressing GFP-labeled microtubule (MT)-associated protein 1 light chain 3 (LC3) (Bampton et al., 2005), an autophagosomal marker that is post-translationally processed into cytosolic LC3-I protein, which is then converted to autophagosomal membrane-associated LC3-II protein (Mizushima et al., 2010). The number of GFP-LC3-positive vesicles is a widely recognized hallmark of autophagy activity within cells (Mizushima et al., 2010). Confocal laser scanning microscopy (CLSM) micrographs (Figure 1A) and determination of GFP-LC3 Relative Immunofluorescence Intensity (RFI)/cell (Figure 1B) in GFP-LC3-expressing HeLa cells treated with Retro-2 showed a time-dependent increase in the number of GFP-LC3-positive vesicles invading the cell cytoplasm relative to untreated cells and DMSO (vehicle of Retro-2)-treated cells. Moreover, quantification of the number of small (5 μm^2^), large (10 μm^2^), and very large (20 μm^2^) GFP-LC3-positive vesicles per cell showed a significant increase over time in the number of large and very large vesicles in Retro-2-treated cells (Figure 1C). Furthermore, the effect of Retro-2 was concentration-dependent, as GFP-LC3 RFI increased in the presence of increasing concentrations of Retro-2 (Figure 1D). Treatment with 1 μM Retro-2 resulted in a large significant increase in the number of small and large vesicles, whereas almost all the vesicles were very large in cells treated with 5 μM Retro-2 (Figure 1E). The amount of LC3-II protein is a second widely recognized hallmark of autophagy (Mizushima et al., 2010). Western-blot analysis of LC3 processing and quantification showed an increase over time of the abundance of LC3-II protein in cells treated with Retro-2 (Figures 1F,G). We next analyzed autophagic flux by treating the cells with the lysosomotropic agent, chloroquine (CQ), which inhibits lysosomal protease degradation of LC3-II (Mizushima et al., 2010). Western blotting and quantification (Figures 1H,I) showed that, as expected, the abundance of LC3-II protein was greater in control cells treated with CQ compared to control untreated cells. In contrast, the abundance of LC3-II protein was not significantly different in Retro-2-treated cells in the presence of CQ from that of Retro-2-treated cells without the inhibitor. This result shows that Retro-2 blocks the autophagic flux.

### Retro-2 Impairs the Trafficking Between Autophagosomes and Lysosomes

**FIGURE 2:**
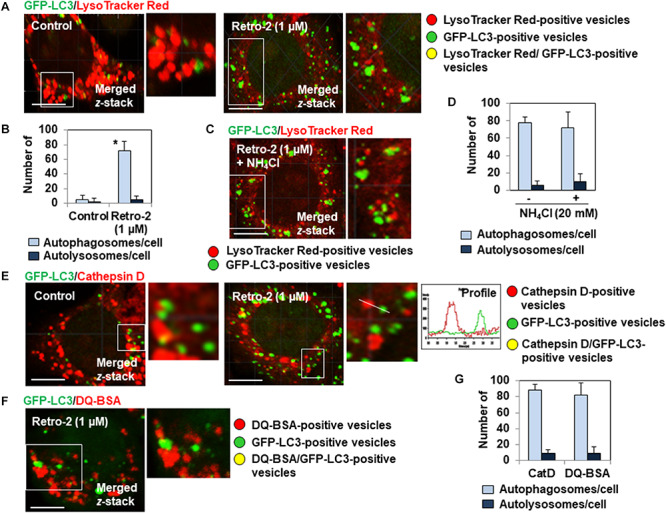
Retro-2 impairs the formation of autolysosomes. **(A)** A representative CLSM micrograph showing the presence of LysoTracker Red-positive vesicles (lysosomes), the rare presence of small GFP-LC3-positive vesicles (autophagosomes), and the absence of GFP-LC3/LysoTracker Red-positive vesicles (autolysosomes) in a control cell. A representative CLSM micrograph showing the presence of lysosomes, the elevated presence of very large autophagosomes, and the absence of autolysosomes in a Retro-2-treated cell (1 μM, 4 h of treatment). **(B)** Graph bar of quantification of numbers of autophagosomes/cell and autolysosomes/cell in cells treated for 4 h with Retro-2 (1 μM). **(C)** A representative CLSM micrograph showing the presence of LysoTracker Red-positive vesicles and GFP-LC3-positive vesicles in a cell treated for 4 h with Retro-2 (1 μM) in the presence of NH_4_Cl (20 mM). **(D)** Graph bar of quantification showed the equal numbers of autophagosomes and the absence of autolysosomes in cells treated for 4 h with Retro-2 (1 μM) in the presence or not of NH_4_Cl (20 mM). **(E)** A representative CLSM micrograph showing the high number of autophagosomes and cathepsin D-positive lysosomes, and the absence of autolysosomes in a Retro-2-treated cell (1 μM, 4 h of treatment). Graph (Profile) showing the absence of colocalization of GFP-LC3 (Green) and Cathepsin D (Red) fluorescent signals measured along the white orientation bar. Pearson’s correlation coefficient was –0.26, indicative of the absence of fusion between autophagosomes and lysosomes. **(F)** A representative CLSM micrograph showing in a Retro-2-treated cell (1 μM, 4 h of treatment) loaded with DQ Red BSA (DeQuenched Bovine Serum Albumin), which emits red fluorescence when it is protease degraded, the presence of red fluorescent-positive vesicles and large GFP-LC3 dots cells, and the absence of vesicles showing a yellow fluorescence resulting of cocalization between DQ Red BSA fluorescence and GFP-LC3 fluorescence. **(G)** Graph bar of quantification in Retro-2-treated cells (4 h of treatment with 1 μM) of numbers of autophagosomes/cell and autolysosomes/cell assessed by observation of Cathepsin D immunolabeling and DQ Red BSA assay. CLMS micrographs are representative of two separate experiments in duplicate. Scale bar, 10 μm. White boxed regions delineate the areas shown in adjacent high-magnification images. Quantification was performed using ImageJ software by examining at least 25 cells per treatment condition in two separate experiments in duplicate. Values represent the average (±SEM). Data were analyzed using the unpaired Student *t*-test. *P < 0.01 compared to Control **(D)**.

We next investigated whether the very large GFP-LC3-positive vesicles observed in the cytoplasm of Retro-2-treated cells have the characteristics of autolysosomes. We thus loaded the GFP-LC3-expressing HeLa cells with LysoTracker Red, a small, highly diffusible, membrane-permeable lysosomotropic molecule that labels the acidic compartments of cells (Mizushima et al., 2010). This allows the identification and quantification of lysosomes (LysoTracker red labeling), autophagosomes (GFP-LC3 green labeling), and autolysosomes (yellow labeling by merged GFP-LC3/LysoTracker Red signals). As expected, we found the strong presence of lysosomes, almost no autophagosomes, and no autolysosomes in control cells (Figures 2A,B). In contrast, Retro-2-treated cells contained many large autophagosomes and no autolysosomes (Figures 2A,B). There was no change in the number of GFP-LC3-positive vesicles in cells treated for 4 h with Retro-2 in the presence of NH_4_Cl, blocking the protease-dependent degradative activity (Xie et al., 2010), compared to cells treated with Retro-2 in absence of NH_4_Cl (Figures 2C,D). By immunolabeling for the detection of lysosomal hydrolase, cathepsin D (Bright et al., 2016), we confirmed the absence of autolysosomes in Retro-2-treated cells. Indeed, confocal observation showed the presence of a large number of autophagosomes and cathepsin D-positive lysosomes and the absence of vesicles positive for yellow fluorescence (GFP-LC3 fluorescence plus cathepsin D fluorescence) demonstrating an absence of autolysosomes (Figures 2E,G). Finally, the lack of degradative character of large GFP-LC3-positive vesicles was evaluated by loading the cells with DQ Red BSA (DeQuenched Bovine Serum Albumin) Red which labeled intracellular degradative compartments (Vazquez and Colombo, 2009). Confocal observation showed the presence in Retro-2-treated cells of lysosomes positive for red fluorescence, the presence of small and large GFP-LC3-positive vesicles and the absence of vesicles positive for yellow fluorescence (GFP-LC3 fluorescence plus DQ Red BSA fluorescence) demonstrating an absence of autolysosomes (Figures 2F,G). Overall, these results show that the accumulation of autophagosomes in the cytoplasm of Retro-2 cells was accompanied by a defect in the formation of autolysosomes.

### Retro-2 Disassembles the Cell MT Network

**FIGURE 3:**
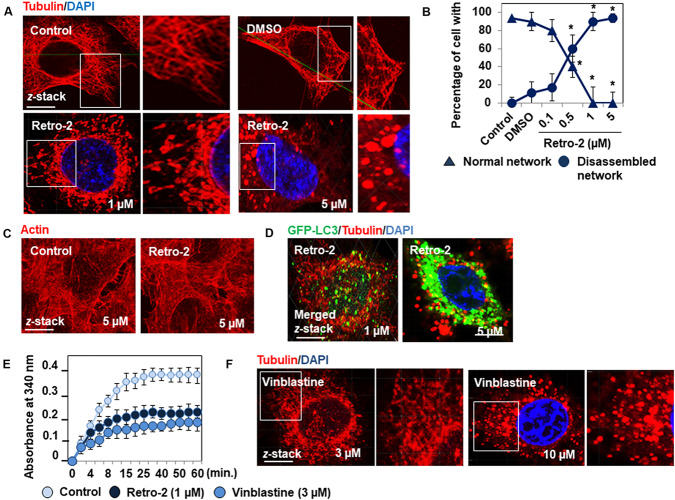
Retro-2 disassembles the MT network of HeLa cells. **(A)** Representative CLSM micrographs showing the well-organized immunolabeled tubulin network in a control cell and the concentration-dependent disassembly of the tubulin network in cells treated with increasing concentrations of Retro-2. Note the concentration-dependent appearance of fragmented, tubulin-positive bar-like structures and tubulin-positive vesicle-like aggregates. **(B)** Quantification of cells expressing well-organized or disassembled tubulin-positive fibers in untreated cells and cells treated with DMSO or increasing concentrations of Retro-2. **(C)** Representative CLSM micrographs showing the actin cytoskeleton in a control cell and in a cell treated with Retro-2 (1 μM). **(D)** Representative CLSM micrographs showing simultaneously tubulin-positive bar-like structures and small GFP-LC3-positive vesicles (1 μM), and very large GFP-LC3-positive vesicles and tubulin-positive vesicle-like aggregates (5 μM) in GFP-LC3-expressing Retro-2-treated HeLa cells. **(E)** Graph showing the Retro-2- and vinblastine-induced inhibition of time-dependent tubulin polymerization *in vitro*. **(F)** Representative CLSM micrographs showing the concentration-dependent disassembly of the tubulin network in cells treated with increasing concentrations of vinblastin. Note the concentration-dependent appearance of fragmented, tubulin-positive bar-like structures and tubulin-positive vesicle-like aggregates. CLSM micrographs are representative of two separate experiments in duplicate. Scale bar, 10 μm. White boxed regions delineate the areas shown in the adjacent high-magnification images. Confocal quantification was conducted by examining at least 30 cells for each condition ImageJ software. *In vitro* tubulin polymerization was measured by spectrophotometry at 340 nm and the data recorded at 30-s intervals for 60 min at 37°C. Values represent the average of two separate experiments in duplicate (±SEM). Data were analyzed using the unpaired Student *t*-test. *P < 0.01 compared to Control **(B)**.

The formation of autolysosomes follows the MT-dependent trafficking of autophagosomes toward lysosomes (Mackeh et al., 2013) followed by their fusion controlled by membrane-associated SNAREs (soluble *N*-ethylmaleimide-sensitive factor attachment protein receptors) platforms (Moreau et al., 2013). Consequently, the lack of autolysosome formation in the presence of Retro-2 prompted us to examine the organization of the MT network in treated cells. Indirect immunolabeling of α-tubulin showed that control cells express the typical straight, fine MT network organized from the cell nucleus to the cell periphery (Figures 3A,B). Cells treated with increasing concentrations of Retro-2 showed extensive disassembly of the MT network (Figures 3A,B). Fragmented tubulin-positive bar-like structures appeared in the cytoplasm at a concentration of 0.5 μM. MT fragmentation was more extensive at a concentration of 1 μM, resulting in large bar-like structures. Only tubulin-positive vesicle-like aggregates randomly dispersed throughout the cell cytoplasm were observed at a concentration of 5 μM. In contrast, Retro-2 did not affect the cell actin cytoskeleton (Figure 3C). We then simultaneously examined the GFP-LC3-positive vesicles, the tubulin-positive bar-like structures, and tubulin-positive vesicle-like aggregates in GFP-LC3-expressing Retro-2-treated HeLa cells. CLSM micrographs showed that the size of the GFP-LC3-positive vesicles increased as the level of MT disassembly increased by transformation of the bar-like structures into vesicle-like aggregates (Figure 3D). These results prompted us to conduct a control experiment to examine whether Retro-2 affects GTP-dependent tubulin polymerization *in vitro*. Purified tubulin polymerized time-dependently to steady state in the presence of GTP, whereas polymerization was inhibited in the presence of Retro-2 (Figure 3E). As control, we showed that the microtubule disassembly agent (MDA) vinblastine disassembled MT network in HeLa cells and inhibited tubulin polymerization *in vitro* (Figures 3E,F) consistent with previous observations (Kochl et al., 2006; Kuznetsov et al., 2009). Overall, these results reveal a hitherto unreported disassembly effect of the small trafficking inhibitor on the cell MT network.

### Retro-2 Abolishes the Cytoplasmic Spreading of GFP-LC3-Positive Vesicles

**FIGURE 4:**
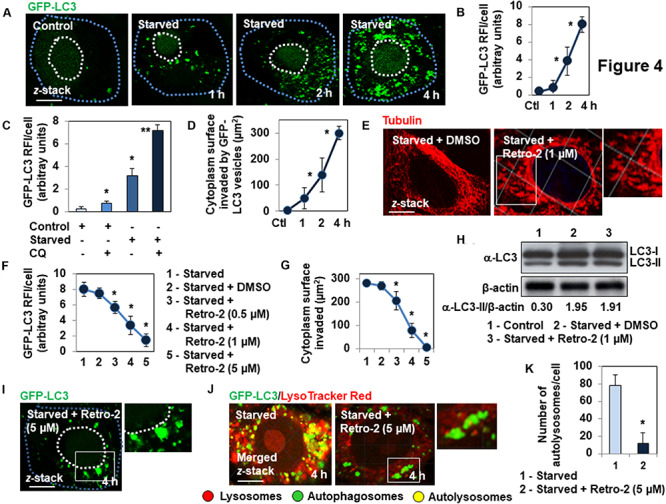
Retro-2 impairs the spreading of autophagic vesicles into the cytoplasm of nutrient-starved GFP-LC3-expressing Hela cells. **(A)** Representative CLSM micrographs showing the time-dependent invasion of the cytoplasm of nutrient-starved cells by GFP-LC3-positive vesicles. **(B)** Quantification of the GFP-LC3 relative immunofluorescence intensity (RFI), showing the increase of GFP-LC3-positive vesicles in nutrient-starved cells over a time-course. **(C)** Quantification of the GFP-LC3 RFI, showing the increase of GFP-LC3-positive vesicles in chloroquine (CQ)-treated Control and Starved cells compared to untreated Control and Starved cells. **(D)** Quantification showing the increase of cytoplasm surface invaded by GFP-LC3-positive vesicles in nutrient-starved cells over a time-course. **(E)** Disassembly of MT network in Retro-2-treated 4 h-nutrient-starved cells. **(F)** Representative CLSM micrographs showing the concentration-dependent decrease of cytoplasmic surface invaded by GFP-LC3-positive vesicles in 4 h-nutrient-starved, Retro-2-treated cells. **(G)** Quantification of the GFP-LC3 RFI showing the concentration-dependent decrease of GFP-LC3-positive vesicles in 4 h-nutrient starved cells treated with increasing concentrations of Retro-2. **(H)** Quantification of the surface of the cell cytoplasm of nutrient-starved Retro-2-treated cells invaded by GFP-LC3-positive vesicles, showing the concentration-dependent decrease of invasion. **(I)** A representative Western blot showing LC3 processing in control cells, 4 h-starved cells, and 4 h-starved cells treated with Retro-2 (1 μM). β-actin was monitored to ensure equal loading of the lanes. **(J)** Representative CLSM micrograph (Left) showing in cells loaded with LysoTracker Red, the elevated presence of autolysosomes (GFP-LC3/LysoTracker Red-positive vesicles) in the cytoplasm of a 4 h-nutrient-starved cell. Representative CLSM micrograph (Right) showing the elevated presence of very large autophagosomes (GFP-LC3-positive vesicles) mostly localizing at the perinuclear domain of a 4 h-nutrient-starved Retro-2 (1 μM)-treated cell, and the absence of cytoplasmic autolysosomes. Pearson’s correlation coefficient was –0.63 for nutrient-starved cells, indicative of classical fusion between the autophagosomes and lysosomes. Pearson’s correlation coefficient was –0.22 for Retro-2-treated nutrient-starved cells, indicative of the absence of fusion between autophagosomes and lysosomes. **(K)** Quantification of numbers of autolysosomes/cell showing their disappearance in the cytoplasm of 4 h-nutrient starved Retro-2-treated cells (1 μM). CLSM micrographs are representative of three separate experiments. Dotted line in white delineates cell nucleus. Dotted line in blue delineates cell cytoplasm. White boxed regions delineate the areas shown in the adjacent high-magnification images. Confocal quantification was performed using ImageJ software. Pearson’s correlation coefficient was calculated using JaCoP ImageJ software. The western blot is representative of two separate experiments in duplicate. Western blot quantification was performed using ImageJ software. Values represent the average (±SEM). Data were analyzed using the unpaired Student *t*-test. *P < 0.01 compared to Control (Ctl) **(B–D,K)**. *P < 0.01 compared to Starved **(G,H)**. **P < 0.01 compared to Starved **(C)**.

We confirmed that Retro-2 affects MT-dependent vacuolar trafficking of autophagosomes using the nutrient-starvation autophagy model (Russell et al., 2014). Nutrient starvation resulted in the time-dependent appearance and invasion of the cell cytoplasm by GFP-LC3-positive vesicles, as shown by microscopy (Figures 4A,B), determination of the number of GFP-LC3 RFI/cell (Figure 4C), and measurement of the cytoplasmic surface invaded by GFP-LC3-positive vesicles (Figure 4D) over time. The cells starved of nutrients for 2 h showed the classical increase of the number of GFP-LC3 vesicles in the presence of CQ compared to untreated nutrients starved cells (Figure 4C). Cells starved of nutrients for 4 h in the presence of Retro-2 showed a disassembled MT network (Figure 4E). Cells starved of nutrients for 4 h in the presence of increasing concentrations of Retro-2 showed a marked concentration-dependent decrease in the cytoplasmic presence of GFP-LC3-positive vesicles (Figures 4F,G). Of note, large GFP-LC3-positive vesicles remained localized within the vicinity of the nucleus when the spreading of GFP-LC3-positive vesicles was entirely abolished by treatment with 5 μM Retro-2 (Figure 4H).Western-blot analysis of LC3 processing showed an identical increase in the abundance of LC3-II protein in nutrient-starved cells, treated or not with Retro-2 (Figure 4I). This was indicating that the decrease of the spreading of GFP-LC3-positive vesicles throughout the cytoplasm of Retro-2-treated cells did not result from the abolition of nutrient starvation-induced autophagy. We next examined the presence of autophagosomes and autolysosomes within untreated and Retro-2-treated nutrient-starved cells by loading the cells with LysoTracker Red. As expected (Mizushima et al., 2010), microscopy and quantification of the number of autophagosomes/cell and autolysosomes/cell showed that a very high number of autolysosomes were present in the cell cytoplasm of untreated nutrient-starved cells (Figures 4J,K). In contrast, the cytoplasm of Retro-2-treated nutrient-starved cells was devoid of autolysosomes (Figures 4J,K). Of note, when the cytoplasm of Retro-2-treated nutrient-starved cells was devoid of autophagosomes, it remained very large autophagosomes localizing mainly to the perinuclear area (Figure 4J). These results show that Retro-2 impairs MT-dependent spreading of autophagosomes through the cytoplasm of nutrient-starved cells in a concentration-dependent manner and abolishes the formation of autolysosomes.

## Discussion

**FIGURE 5:**
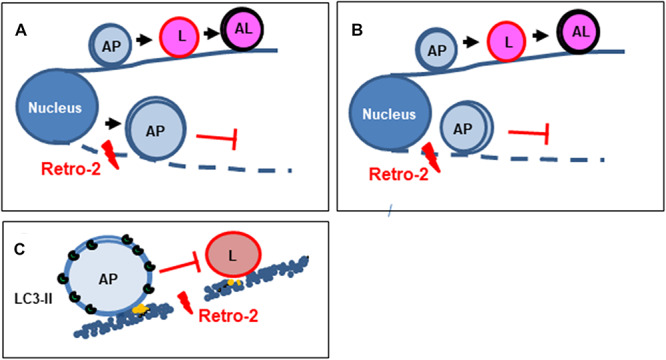
Diagram summarizing the mechanism by which the small trafficking inhibitor Retro-2 modulates MT-dependent vacuolar trafficking in autophagy. **(A)** Autophagosomes normally move toward lysosomes in a MT-dependent manner to form autolysosomes. By disrupting the MT network, Retro-2 impairs the trafficking of autophagosomes and promotes the cytoplasmic accumulation of very large autophagosomes. **(B)** Cell nutrient starvation activates the formation of autophagosomes, which spread throughout the cell cytoplasm in an MT-dependent manner. By disrupting the MT network, Retro-2 blocks such spreading of autophagosomes and induces perinuclear accumulation of very large autophagosomes. **(C)** By disrupting the MT network, Retro-2 impairs the trafficking-dependent formation of autolysosomes. AP, autophagosome; L, lysosome; AL, autolysosome.

We show that the mechanism by which Retro-2 affects vacuolar movement in the autophagy pathway is through the hitherto unsuspected disassembly of the cellular MT network (Figures 5A–C). Four sites on tubulin, called the colchicine, vinca, maytansine, and pironetin sites, are targeted by MDAs (Steinmetz and Prota, 2018). Interestingly, the concentration-dependent morphological changes of MTs induced by Retro-2 resemble the concentration-dependent formation of tubulin-positive curved fragments and aggregates/paracrystals triggered by vinblastine (Kochl et al., 2006). Future experiments are needed to determine whether Retro-2 binds to the vinca site of the tubulin dimer.

We show that Retro-2 strongly initiates autophagy accompanied by the marked cytoplasmic accumulation of very large autophagic vacuoles in nutrient-fed cells (Figure 5A). Similar cytoplasmic accumulation of large autophagic vacuoles has been observed in nutrient-fed cells treated with the MDA agent vinblastine (Xie et al., 2010). The question arises as to how Retro-2 induces autophagy in the fed condition. One possibility is that Retro-2-triggered disassembly of the MT network generates a cellular stress signal, given the essential role of the MT network for cellular homeostasis (De Forges et al., 2012). It is also plausible that it is linked to endoplasmic reticulum (ER) stress. The ER is a ubiquitous subcellular compartment of eukaryotic cells, consisting of a continuous cytoplasmic network of membranous sheets and tubules that play key roles in cellular homeostasis, metabolism, and cellular organization and signaling (Wilkinson, 2019). The autophagy stress response is upregulated following ER stress in several cell types to alleviate triggered deleterious cellular effects (Rashid et al., 2015). Interestingly, under ER stress, autophagosomes (Ganley et al., 2011; Nakashima et al., 2019) and multivesicular bodies (MVBs) (Kanemoto et al., 2016) accumulate in the cell cytoplasm. Retro-2 could create ER stress linked to its recently demonstrated effect on an ER-associated function (Morgens et al., 2019; Forrester et al., 2020). Indeed, Retro-2 inhibits the ASNA1-mediated ER targeting and insertion of tail-anchored proteins (Morgens et al., 2019). Moreover, it blocks delivery of newly synthesized tail-anchored proteins to the ER-targeting factor Asna1, which results in decreased abundance of the Qa-SNARE syntaxin5 (Stx5) at the Golgi (Forrester et al., 2020). An autophagy stress response is activated when Stx5 is silenced at the ER-Golgi (Renna et al., 2011). Interestingly, the small trafficking inhibitor ABMA, which blocks vacuolar trafficking (Wu et al., 2017), like Retro-2 (Gupta et al., 2017), has been shown to induce autophagy and the cytoplasmic accumulation of large MVB/LE-like vacuoles and amphisome-like vacuoles devoid of degradation activity in the nutrient-fed condition (Wu et al., 2020).

We found that Retro-2 decreased the spreading of autophagosomes from the nucleus toward the cell cytoplasm in cells in which autophagy was induced by nutrient starvation and that large autophagic vacuoles remained localized near the nucleus when such spreading was entirely abolished (Figure 5B). Such perinuclear accumulation of large autophagic vacuoles has been observed in nutrient-starved cells treated with the MDA agents vinblastine (Munafo and Colombo, 2001, 2002; Kochl et al., 2006; Fader et al., 2008; Zhou et al., 2013) and nocodazole (Reunanen et al., 1988; Aplin et al., 1992; Webb et al., 2004; Fass et al., 2006; Kochl et al., 2006; Fader et al., 2008). Moreover, similar accumulation of perinuclear clusters of autophagosomes occurs in nutrient-starved cells after depletion of the functional kinesin-1/FYCO1 motor (Cardoso et al., 2009; Pankiv et al., 2010).

We present evidence that the cytoplasmic accumulation of large autophagosomes in the cytoplasm of nutrient-fed Retro-2-treated cells and at vicinity of nucleus of nutrients starved cells is the consequence of the abolition of autolysosome formation (Figure 5C). The formation of this final autophagic degradative vacuolar compartment occurs by the MT-dependent trafficking of autophagosomes toward lysosomes (Jahreiss et al., 2008; Kimura et al., 2008; Longatti and Tooze, 2009). Indeed, autophagosomes move along MT tracks in two directions as a result of the opposing activities of the minus-end-directed motor dynein/dynactin and plus-end-directed motor kinesin/FYCO1 (FYVE and coiled-coil domain-containing protein 1 (Kimura et al., 2008; Cardoso et al., 2009; Zhao and Zhang, 2019). Following MT-dependent movements, the interaction between autophagosomes and lysosomes leads to vacuolar membrane fusion events requiring the engagement of membrane-associated SNAREs-containing fusion platforms (Furuta et al., 2010; Itakura et al., 2012; Matsui et al., 2018; Zhao and Zhang, 2019). Given that Retro-2 exerts Qa-SNAREs Stx5-dependent effects (Stechmann et al., 2010; Canton and Kima, 2012; Nonnenmacher et al., 2015; Morgens et al., 2019; Forrester et al., 2020), it is possible that Retro-2 inhibits the fusion between autophagosomes and lysosomes via an Stx5-dependent mechanism. However, this SNAREs is not present in the committed SNAREs platforms involved in autophagic vacuolar fusion events [Qb-SNARE Vti1b/R-SNARE Vamp8; Qa-SNARE Stx17/Qa-Synaptosomal-Associated protein 29 (SNAP29)/R-SNAREs VAMP7/8; and Stx7/SNAP29/YKT6] (Furuta et al., 2010; Itakura et al., 2012; Jia et al., 2017; Matsui et al., 2018). Thus, we investigated wether the observed effects of Retro-2 on autophagy and vacuolar trafficking occur upstream of the vacuolar fusion events, i.e., at the level of MTs, which ensure the movement of autophagosomes (Jahreiss et al., 2008; Kimura et al., 2008; Longatti and Tooze, 2009). The uncovering of disassembly of the MT network by Retro-2, together with the observation of its effects on autophagy and vacuolar trafficking identical to those previously reported for the MDA agents nocodazole and vinblastine, make it likely that it is through this mechanism that Retro-2 abolishes the interaction between autophagosomes and lysosomes, both in nutrient-fed and nutrient-starved cells.

Cytoplasmic accumulation of autophagosomes elicits cytotoxicity (Button et al., 2017). Autophagic vacuoles accumulated in the cytoplasm of Retro-2-treated cells at concentrations that are much lower than those that induce cytotoxicity (Stechmann et al., 2010). It cannot be excluded that the accumulation of such autophagic vacuoles are an early sign of cytotoxicity. It will thus be important in future experiments to determine whether the Retro-2 induction of autophagy, arrest of the autophagy flux, and impairment of autophagic vacuole maturation contribute or not as a function of concentration to the dynamics of a cell-death mechanisms, including autophagy-mediated cell death (Galluzzi et al., 2018).

Retro-2 has been shown to protect cells against the deleterious and lethal effects of plant ricin and bacterial Shiga toxins by arresting their cellular trafficking in the early endosome (EE)-*trans*-Golgi network (TGN) interface through an Stx5-dependent mechanism and then blocking their trafficking back to the endoplasmic reticulum (ER) (Stechmann et al., 2010). Moreover, Retro-2 decreases adenovirus 2 transduction and Stx5-dependent transport to the Golgi (Nonnenmacher et al., 2015). Recent elegant studies have surprisingly revealed that Retro-2 indeed affects ER functions, which in turn affects trafficking events at the EE/TGN interface (Morgens et al., 2019; Forrester et al., 2020). However, observations that Retro-2 blocks the entry of several viruses at the cell membrane (Dai et al., 2018; Shtanko et al., 2018), in which Stx5 is not expressed, suggest that the small compound additionally exerts Stx5-independent inhibitory effects at sites other than the EE/TGN and ER. Our results reveal that the MT network is a new ER/Golgi-independent cellular target of Retro-2. This raises several questions regarding that Retro-2 does not affect certain endogenous traffickings essential for cellular homeostasis (Stechmann et al., 2010). The same question arises regarding the effect of Retro-2 at the ER. As recently underlined (Farhan, 2020), it is difficult to understand why the effect of Retro-2 on a general Sec16A regulator of ER export at ER exit sites (Morgens et al., 2019; Forrester et al., 2020) does not exert negative pleiotropic effects, given that the ER is essential for cellular homeostasis (Sicari et al., 2020). Interestingly, ER exit sites proximal to the phagophore assembly site are landmarks for the assembly of the ATG machinery of autophagy (Graef et al., 2013). It has been postulated that there may be specific states or pools of Sec16A and that one of these is selectively targeted by Retro-2 (Farhan, 2020). For the effect of Retro-2 on MT the network, it is possible that Retro-2 selectively affects various cellular pools of MTs (Janke and Bulinski, 2011) or one or several existing stable and dynamic sub-populations of αβ-tubulin dimers, stable MTs with numerous post-translational modifications playing particular roles in traffic of autophagic vacuoles (Geeraert et al., 2010; Xie et al., 2010; Mohan et al., 2019). These create interesting opportunities for future studies.

Our finding and reports by Wu et al. (2019, 2020) offer new perspectives for Retro-2 and related compounds because autophagy is a druggable target (Morel et al., 2017) and small autophagy-regulating compounds are under experimental and clinical investigation to combat diverse diseases, including cancer (Galluzzi et al., 2017).

## Materials and Methods

### Reagents and Antibodies

Retro-2 [2,3-Dihydro-2-(5-methyl-2-thienyl)-3-phenyl-4(1H)-quinazolinone], vinblastine and rabbit anti-actin (A2066) were purchased from Sigma-Aldrich. LysoTracker Red^®^, DQ^TM^ Red BSA, and 4′,6-diamidino-2-phenylindole (DAPI) were from Invitrogen Life Technologies. Anti-α-tubulin mAb (11H10) was from Cell Signaling Technology (Ozyme, Saint Quentin en Yvelines, 78053, France). Anti-actin (A2066) was from Sigma-Aldrich (Saint Quentin Fallavier, 38070, France). Anti-human cathepsin D (E-7) was from Santa Cruz Biotechnology Inc. (CliniSciences, Nanterre 92000, France). Anti-LC3 antibody (L7543), anti-β-actin-peroxydase (A3853), goat anti-rabbit IgG- and sheep anti-mouse IgG-peroxidase antibodies (A0545 and A5906, respectively) were from Sigma-Aldrich. Appropriate secondary antibodies for indirect immunofluorescence labeling were purchased from Jackson Immunoresearch Laboratories, Inc. (West Grove, PA, United States) and Molecular Probes Inc. (Invitrogen Life Technologies).

### Cell Line, Culture Conditions, and Treatments

HeLa cells and HeLa cells stably transfected with rat green fluorescent protein (GFP)-LC3 (GFP-LC3-expressing HeLa cells) were kindly provided by A. M. Tolkovsky (Department of Biochemistry, University of Cambridge, United Kingdom) (Bampton et al., 2005). Cells were seeded and grown in culture plates (TPP, ATGC Biotechnologie, Noisy Le Grand, France) containing coverslips and cultured in RPMI-1640 with L-glutamine (Life Technologies, Cergy, France) at 37°C in an atmosphere containing 5% CO_2_. For autophagy induction by amino-acid deprivation (Mizushima et al., 2010), the cells were incubated for the indicated times in the presence of Earle’s balanced salt solution (EBSS) (Sigma) at 37°C in an atmosphere containing 5% CO_2_. Before being processed, the cells were washed twice with phosphate-buffered saline (PBS). For treatments, the cells were incubated continuously with or without the compounds.

### Immunofluorescence Labeling

For indirect immunofluorescence labeling cells were cultured in 24-well TPP tissue culture plates (ATGC, Marne-la-Vallée, France) with coverslips. Specimens were fixed with 3% paraformaldehyde in PBS for 5 min at room temperature, washed three times with PBS, treated for 10 min with PBS containing 50 mM NH4Cl to neutralize the aldehyde, and blocked by adding PBS containing 0.2% gelatin. Cells were permeabilized by incubation with 0.2% Triton X-100 in PBS for 4 min at room temperature and then washed three times with PBS. Cellular MTs and the actin cytoskeleton were immunolabeled with anti-α-tubulin (1/500) and anti-actin (1/100), respectively. To visualize the cell nucleus, cells were stained for 15 min with 100 μg/ml DAPI (Invitrogen Life Technologies). Coverslips were mounted using Dako fluorescent mounting medium (Invitrogen Life Technologies).

### LysoTracker^®^ Red Labeling and DQ^TM^ Red BSA Assay

Lysosomes and autolysosomes were labeled by incubating the cells with LysoTracker^®^ Red (50 nM) for 60 min. To monitor lysosomal activity, cells were incubated with DQ^TM^ Red BSA (10 μg/ml) for 2 h. The labeled cells were washed three times with sterile PBS and then fixed with 3% paraformaldehyde in PBS for 5 min at room temperature. Coverslips were mounted using Dako fluorescent mounting medium.

### Confocal Scanning Immunofluorescence Microscopy and Imaging Analysis

Samples were imaged with an inverted g-STED TCS SP8 Leica confocal microscope (Leica, Germany) using a HC PL APO CS2 63×/1.40 oil immersion objective lens. The instrument was equipped with a 405 nm diode for DAPI excitation and a WLL Laser (495 nm excitation wavelength for GFP and 552 nm for rhodamine). Blue, green, and red fluorescence emission were collected using 420– 460-, 505– 550-, and 560–760-nm wide emission slits, respectively, in sequential mode. The pinhole was set at 1.0 Airy unit, giving an optical slice thickness of 0.89 μm. Twelve-bit numerical images were processed using Leica SP8 LAS X software (Version 2.0.1; Leica, Germany). Stacks of images were collected every 0.30 μm along the *z*-axis. Since the vesicles are 3-dimensional specimens, optical sectioning was done along the *x–z* plane to get a *z* stack for each specimen. The methods used for analysis of CLSM micrographs have been previously described (Lievin-Le Moal et al., 2011). Confocal *z*-stack images were used to measure the defined surface or the RFI/cell. A smoothing Gaussian filter of 0.09 was applied before thresholding. The “segmented line” tool of ImageJ software (version 1.42, NIH) was used to delineate a surface of interest in each CLSM micrograph analyzed. The “Plot profile” tool of ImageJ was used to obtain the intensity profile. Imaris Measurement ImageJ was used to quantify the surface of cell cytoplasm invaded by GFP-LC3-positive vesicles. Determination of the size of GFP-LC3 vesicles in a single cell was conducted using Imaris Measurement Pro software. The number of GFP- LC3-, LysoTracker-, and GFP-LC3/LysoTracker-positive vesicles in a single cell were quantified using ImageJ software. Pearson’s coefficient, indicating colocalization or not of the GFP-LC3 signal with the LysoTracker Red fluorescent or cathepsin D immunofluorescent signals, was calculated using JaCoP ImageJ software. Quantitative values obtained were exported to Excel for further analysis and graphical representation. All imaging analyses and quantifications were performed blindly to eliminate any possible bias. Images were resized using Adobe Photoshop CS4 (San Jose, CA, United States).

### Western-Blot Analysis

Cells are washed once with cold PBS and then treated for 15 min at 4°C with extraction buffer (25 mM Hepes, 0.5% Triton, 150 mM NaCl, and 2 mM EDTA) containing proteases and phosphatase inhibitors. Protein fractions were dissolved in the appropriate volume of Laemmli buffer and heated at 100°C for 5 min. The proteins were immediately separated on 12% SDS-polyacrylamide gels. For western-blot analysis, gels were transferred to polyvinylidene difluoride membranes (PerkinElmer, Les Ulis, France). A primary rabbit anti-LC3 antibody was used to reveal LC3-I and LC3-II proteins. A primary mouse antibody specific to β-actin was used to verify the equal loading of lanes. Primary antibodies were revealed with anti-rabbit- or anti-mouse-peroxidase secondary antibodies. The abundance of LC3-II and β-actin proteins in the western blots was quantified by densitometry using ImageJ software.

### Analysis of Tubulin Polymerization *in vitro*

Compounds were tested using the Tubulin Polymerization BK004P kit as specified by the manufacturer (Cytoskeleton, Inc., Denver, CO, United States). Briefly, tubulin protein (>99% purity) was resuspended (300 μg/sample) in 100 μl G-PEM buffer (80 mM PIPES, 2 mM MgCl_2_, 0.5 mM EGTA, 1.0 mM GTP, and pH 6.9) plus 5% glycerol, with or without the test compound, at 4°C. Then the sample mixture was transferred to a pre-warmed 96-well plate and polymerization measured by the change in absorbance at 340 nm every 1 min for 45 min (SpectraMAX Plus; Molecular Devices, Inc., Sunnyvale, CA, United States) at 37°C.

### Statistical Analysis

Data are reported as the mean ± standard error of the mean (SEM) of three independent experiments in duplicate. All graphs were produced using Microsoft Excel software. Comparisons between the experimental groups were performed using the unpaired Student *t*-test. Significance was established when *P* < 0.01.

## Data Availability Statement

The datasets generated for this study are available on request to the corresponding author.

## Author Contributions

VL-L: conceptualization, resources, and writing – original draft. VL-L and VN: methodology, imaging study, data analysis, review, and editing.

## Conflict of Interest

The authors declare that the research was conducted in the absence of any commercial or financial relationships that could be construed as a potential conflict of interest.
